# Comparison of echocardiographic (US) volumetry with cardiac magnetic resonance (CMR) imaging in transfusion dependent thalassemia major (TM)

**DOI:** 10.1186/1476-7120-5-24

**Published:** 2007-07-14

**Authors:** Anastasios Giakoumis, Vasilis Berdoukas, Efstathios Gotsis, Athanassios Aessopos

**Affiliations:** 1 First Department of Internal Medicine of Laiko General Hospital, University of Athens, Athens, Greece; 2 Department of Pediatrics, University of New South Wales, Sydney, Australia; 3 MRI Department, Euromedica "Enkephalos", Athens, Greece

## Abstract

**Background:**

Despite advances in survival in patients with thalassemia major (TM) the most common cause of death is cardiac disease. Regular cardiac follow-up is imperative in order to identify and reverse pathology. Cardiac Magnetic Resonance (CMR) and Echocardiography (US) are applied in parallel to TM patients for cardiac evaluation and ongoing monitoring. A comparison between mutual features would be useful in order to assess the accuracy and reliability of the two methods, with a particular focus on routine US application. TM's special attributes offer an excellent opportunity for cardiac imaging research that has universal general purpose applications.

**Methods:**

135 TM patients underwent US (Teichholz's M-mode formula – rapidly accessible means of measuring volumes and ejection fraction) and CMR volumetry. Paired-samples t-test, Passing & Badlock regression and Bland & Altman plot were used while comparing the common parameters between the CMR and the US.

**Results:**

We found that the US volumes were underestimated, especially the end-diastolic volume (p < 0.001). The end-systolic volume showed a borderline two-tailed probability (p ≈ 0.05). The correlation for the ejection fraction was acceptable (r = 0.60) without a statistically significant difference (p = 0.37) and the Bland Altman plot range was narrow (25.8%). There was a satisfactory correlation of the US' shortening fraction with CMR's ejection fraction (r = 0.58).

**Conclusion:**

In cases where cardiac wall movement abnormalities are absent, the US Teichholz's M-mode formula for volume measurements, though less sophisticated in comparison to the high resolution CMR technique, offers an adequate ejection fraction estimation for routine use, especially when monitoring gross alterations in cardiac function over time, and is easy to perform.

## Background

Patients with homozygous, transfusion dependent thalassemia (TM) exhibit a broad spectrum of cardiac pathology due to the nature of their disease. Methods of cardiac imaging, such as the US and CMR, assess cardiac function in thalassemia major and estimate the extent of damage. Having in mind the special attributes of this patient population and their need for frequent evaluation of heart performance and cardiac iron load with US and CMR [1], TM offers an excellent opportunity for cardiac imaging research that has universal general purpose applications.

Echocardiography (US) is a very useful examination since its application is widespread, safe, economical and very commonly used in every day medical practice. However the image acquisition depends on the operator and the acoustic window [2]. Reproducibility is reasonable in normal ventricles [3], but the quantifications of volumes and mass rely on geometrical assumptions that do not apply to ventricles undergoing asymmetric cardiac remodelling such as in cardiomyopathy [4,5] (presence of asynergy and dilatation).

The most recent advance in cardiac imaging is with Cardiac Magnetic Resonance (CMR). Like echocardiography, it is free of ionizing radiation, non-invasive and reliable. In addition it's independent of geometric assumptions [6,7] and has been shown to be accurate [8,9] and reproducible [10-19]. However, unlike echocardiography, it is expensive (up to over 5 times the cost of US) [20,21], time consuming, performed within a claustrophobic environment and cannot be used in patients with cardiac pacemakers or metallic implants in their upper torso. It is also not universally available.

Considering the above, a comparison between mutual features would be useful in order to assess the accuracy and reliability of the two methods, with a particular focus on routine US application. From the common measured left ventricle (LV) parameters in both techniques the end-diastolic volume (EDv), the end-systolic volume (ESv), the stroke volume (Sv) and, most importantly, the ejection fraction (EF) are very important in assessing the overall heart performance and help classify patients in condition related risk groups.

## Methods

One hundred and thirty five (135) patients with TM attending an outpatient clinic, regularly transfused at approximately two weekly intervals and with regular chelation therapy, were assessed with CMR and had ultrasound cardiac assessment within an interval of 1 month from their CMR (prior or later). There were 72 females and 63 males with a mean age of 29.96 (± 5.91) years. The mean body surface area was 1.66 (± 0.17) m^2 ^and the mean heart rate was 78.81 (± 10.34) beats per minute. Patients in whom cardiac medication was introduced between studies were excluded from the comparison analysis, due to the possible influence with either study's results.

### Echocardiography (US)

Complete M-mode, two-dimensional and Doppler (pulsed-wave, continuous-wave and color) echocardiography was performed at rest, using an Aloca ProSound SSD 5500 ultrasound system (Aloca Co., Tokyo, Japan). Patients were examined in the mid-interval between two scheduled transfusions. The same applied for the CMR study. All echocardiographic and Doppler studies were carried out by the same observer (AA). Chamber dimensions were measured according to the recommendations of the American Society of Echocardiography (ASE) [22] using M-mode echocardiography, while two-dimensional echo was used wherever M-mode measurements were considered unreliable [23,24]. Stroke volume, ejection fraction and cardiac output estimation were based on the calculation of LV end-diastole and end-systole volumes with Teichholz's formula, based on the most accurate and easily performable M-mode measurement algorithm [4,5]:

V = [7/(2.4 + LVId)]·[LVId]^3^, with LVId being the LV internal dimension (end-diastole and end-systole respectively).

### Cardiovascular Magnetic Resonance (CMR)

A cardiac-dedicated 1.5 Tesla MR imager (Signa CVI, General Electric, Milwaukee, USA) with 40 mT/m gradients, phase array-cardiac rf coil and appropriate cardiac software was used for the CMR measurements. Cardiac volumes and ejection fractions were calculated offline in GE's "Advantage Windows" Workstation using GE's proprietary software MASS_PLUS. GE's algorithm for calculating cardiac volumes is based on a 3D method (in essence the drawn area of endocardium and exocardium of each short-axis slice multiplied by slice thickness and stacked together to create a 3-dimensional object). Additional imaging parameters included a single excitation, a matrix of 224 × 224, bandwidth of 125 kHz, slice thickness of 8 mm, and electrocardiogram (ECG) gating. Each slice in 2-chamber, 4-chamber and short-axis view was acquired in a single breath-hold (15–20 sec), with a 20 phases per cardiac cycle. Cardiac volumes, and thereby ejection fraction and stroke volume, were assessed from 10–12 short-axis slices from the base to the apex of the heart. Although automation worked quite well, manual corrections had to be made occasionally in order to ensure the accuracy of the measurements. All measurements were performed by the same operator (EG). The unit was validated through the Brompton site with five patients who had CMR, once in Athens, then London then again in Athens all within a three week period and showed that the reproducibility was within a 5% confidence [25]. In sum, the 3D-Reconstruction volume calculation formula could be outlined as follows:

V = (A_1 _+ A_2 _+ A_3 _+ ... + A_n_) × 8 mm, with A_1 _to A_n _being the area of each of the 10–12 short-axis slices

### Statistical analysis

Statistical analysis was performed with MedCalc™ (MedCalc Software, Mariakerke, Belgium) using paired-samples t-test, Passing & Badlock regression and Bland & Altman plot when comparing the common features between the CMR and the US. The p values were regarded as significant when <0.05. Continuous variables are expressed as mean ± 1 SD. For correlation, r > 0.5 was considered significant.

The Institutional Review Board approved the study and all subjects signed written consent for the anonymous publication of their data.

## Results

Table 1 demonstrates CMR (a) and US (b) LV parameters of the patients with mean values and standard deviations.

**Table 1:** CMR (a) and US (b) parameters of the patients with mean values and standard deviations.

**a) CMR LV parameters**	**Mean ± SD**
MREDv – End-Diastolic volume (ml)	138.02 ± 42.98
MRESv – End-Systolic volume (ml)	45.51 ± 21.05
MRSv – Stroke volume (ml)	92.51 ± 29.69
MREF – Ejection fraction (%)	67.09 ± 7.88

**b) US LV parameters**	**Mean ± SD**

USEDv – End-Diastolic volume (ml)	123.98 ± 26.48
USESv – End-Systolic volume (ml)	41.05 ± 14.59
USSv – Stroke volume (ml)	82.70 ± 16.51
USSF – Shortening fraction (%)	37.38 ± 4.80
USEF – Ejection fraction (%)	66.87 ± 6.39

Table 2 demonstrates the CMR/US methods comparison by Passing & Badlock regression, paired samples t-tests and Bland & Altman (BA) plot. The best correlation was with the net volumes (r from 0.78 to 0.81). However, the US volumes were relatively underestimated, especially the end-diastolic volume. This explains the statistically significant difference (paired samples t-test) for the end diastolic volume (MREDv – USEDv, p < 0.001), whereas the end systolic volume showed a borderline two-tailed probability (MRESv – USESv, p ≈ 0.05). The correlation for the ejection fraction was acceptable (MREF – USEF, r = 0.60) without a statistically significant difference (t-test p = 0.37) and the Bland Altman plot range was narrow (25.8%). This is explained by the relatively equivalent underestimates of the net volumes. Equally satisfactory was the correlation of the US' shortening fraction (USSF) with CMR's ejection fraction (MREF) (r = 0.58).

**Table 2:** CMR/US methods comparison by Passing & Badlock regression analysis, paired samples t-tests and Bland & Altman (BA) plot.

**CMR – US**	**EDv (ml)**	**ESv (ml)**	**Sv (ml)**	**EF (%)**	**EF – SF (%)**
Mean diff ± SD	11.22 ± 26.03	2.80 ± 12.41	8.25 ± 19.79	0.59 ± 6.60	-
p (t-test)	0.0003	0.0514 (**NS**)	0.0005	0.3684 (**NS**)	-
r (p < 0.0001)	0.8135	0.8081	0.7763	0.5990	0.5771
BA limits	-39.8 to 62.2	-21.5 to 27.1	-30.5 to 47.0	-12.3 to 13.5	-
BA range	102.0	48.6	79.7	25.8	-

**Figure 1:**
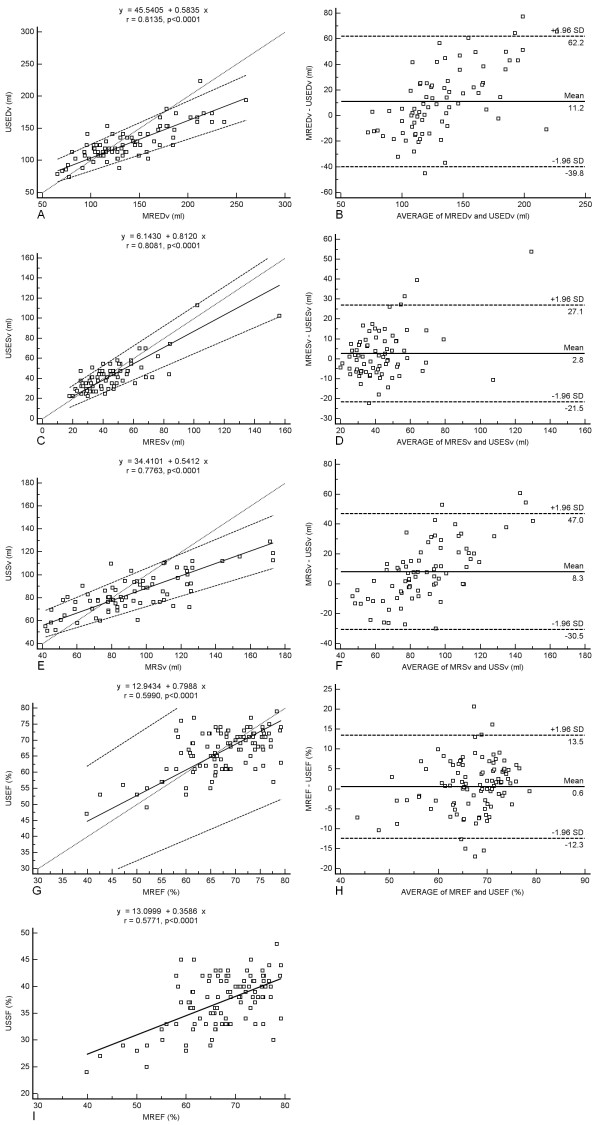
1A-1H. Passing & Badlock scatter diagrams and regression lines, and Bland & Altman plots for the compared parameters. 1I. Scatter diagram and regression line for the USSF-MREF correlation. USEDv: US LV End-Diastolic volume. MREDv: CMR LV End-Diastolic volume. USESv: US LV End-Systolic volume. MRESv: CMR LV End-Systolic volume. USSv: US LV Stroke volume. MRSv: CMR LV Stroke volume. USEF: US LV Ejection fraction. MREF: CMR LV Ejection fraction. USSF: US LV Shortening fraction.

Figures 1A, 1C, 1E and 1G show the Passing & Badlock scatter diagrams and regression lines for the paired parameters. Figures 1B, 1D, 1F and 1H are the Bland & Altman plots for the paired parameters. Figure 1I shows the scatter diagram and regression line for USSF correlated to MREF. The Cusum test in all Passing & Badlock regression analyses showed no significant deviations from linearity.

## Discussion

For this study we used information from a large number of patients (135 in total) who had undergone a CMR examination for follow-up on their cardiac iron overload (T2* score) in parallel to their routine echocardiogram. The US technique employed by our research team did not implement latest technological advances and measurements were calculated with a relatively simple algorithm in order to evaluate one of the most common and rapidly accessible means of measuring EF via US against the high resolution CMR technique.

Our study demonstrated a mild discrepancy of values in the net volume (diastolic and systolic) comparison, with the US derived measurements being relatively and systematically underestimated. However this consistent underestimation was proportionally equal within the diastolic and systolic volume calculations leading to extremely similar ejection fractions between the two methods.

In two studies by Bellenger et al [26,27] where a comparison of left ventricular function measurements was made in patients (21 and 12 respectively) who had undergone orthotopic cardiac transplantation, results demonstrated statistically significant difference with systematic US overestimation of the ejection fraction while comparing CMR to US in both studies (t-test 2.7 ± 6.3% with p < 0.01 in the first study and t-test 7.7 ± 5.9% with p < 0.001 in the second). A poor correlation for the same parameter was also observed (r = 0.41 and r = 0.3 respectively). Regarding the obtained BA plots for the ejection fraction, both studies showed ranges similar to ours (24.6% and 24% accordingly), but the limits defining these ranges were more asymmetric in comparison to ours (-9.6 to 15% and -4 to 20% respectively). The contrast in findings between our study and the previous two may be due to the use of a different technique while assessing cardiac volumes with US (Teichholz's vs. cube formula [28]), the relative disproportion in sample size (135 vs. 12) and/or the diversity of each study's patients' age (young vs. older adults) and heart condition (iron-infiltrative disease vs. immunologic response and heart remodelling following cardiac transplantation). In the present study, the patients were relatively young, thus a good acoustic window was achieved during their US evaluation. In addition, none of them suffered from segmental hypokinesias of the myocardium, minimizing the possible bias that such alterations might introduce in the comparison analysis. It is essential for the Teichholz formula that such cardiac wall movement disorders are not present and a good acoustic window is obtained.

An earlier study by the same research team [29] in a cardiovascular clinic compared ejection fraction measurements via CMR and US (using both the cube and Teichholz formulae). Each of the two paired technique comparisons involved 22 patients. The correlation between CMR and both US techniques was 0.6. For CMR versus the cube and Teichholz formulae the BA limits were wide (-29.9 to 17.6% with a range of 47.5% and -20.6 to 23.3% with a range of 43.9% respectively). Additionally, the cube formula overestimated the ejection fraction (6.1 ± 12.1%) and differed with a statistical significance from CMR (t-test p < 0.05), while on the other hand Teichholz's formula underestimated the same parameter (1.3 ± 11.2%) and t-test showed no statistically significant difference from CMR. Our study's outcomes accord the results of the latter comparison (CMR – Teichholz) and furthermore demonstrate a better agreement between CMR and US (BA range of 25.8% vs. 43.9%). Therefore Teichholz's easy-to-use formula is definitely sufficient for the everyday practice. This fact allows echocardiography, despite the slight underestimation observed, to assume a particularly useful role in cases where a reliable determination of EF is promptly needed, and provided that cardiac wall motility is normal.

In the present study, due to patients' intolerance to multiple evaluations and significant examination cost, particularly CMR's, we were unable to perform an interstudy reproducibility analysis of both methods. We do believe however that this represents a small limitation to this work.

## Conclusion

The good correlation and the lack of statistically significant difference between the measurements of the two techniques for ejection fraction indicate that both methods are accurate and interchangeable for that assessment. In cases where application requirements described are met, the US Teichholz's M-mode formula for volume measurements, though less sophisticated in comparison to the high resolution CMR technique, offers an adequate EF estimation for routine use, especially when monitoring gross alterations in cardiac function over time, and is easy to perform, in contrast to the not always readily available CMR technique.

## Competing interests

With respect to conflicts of interest, Professor Aessopos has received travel assistance for conferences from Novartis Hellas and Demos and Dr. Berdoukas is a consultant for ApoPharma Inc. (Canada) and holds a confidentiality agreement with Novartis for the development of the drug Exjade.

Dr. Giakoumis and Dr. Gotsis declare that they have no competing interests.

## Authors' contributions

AG conceived and designed the study, carried out the acquisition and statistical analysis of data and drafted the manuscript.

VB assisted in the statistical analysis of data, interpretation of results and manuscript drafting.

EG carried out the CMR volumetry study.

AA carried out the US volumetry study, interpreted results, revised the manuscript for important intellectual content and coordinated the whole process.

All authors read and approved the final manuscript.
